# CryoFSL: an annotation-efficient, few-shot learning framework for robust protein particle picking in cryo-electron microscopy micrographs

**DOI:** 10.1093/bib/bbag285

**Published:** 2026-06-05

**Authors:** Biplab Poudel, Rajan Gyawali, Ashwin Dhakal, Jianlin Cheng, Dong Xu

**Affiliations:** Department of Electrical Engineering and Computer Science, NextGen Precision Health, University of Missouri, 416 South 6th Street, Columbia, MO 65211, United States; Bond Life Sciences Center, University of Missouri, 1201 Rollins Street, Columbia, MO 65211, United States; Department of Applied Computing, Lander University, 320 Stanley Avenue, Greenwood, SC 29649, United States; Department of Electrical Engineering and Computer Science, NextGen Precision Health, University of Missouri, 416 South 6th Street, Columbia, MO 65211, United States; Department of Electrical Engineering and Computer Science, NextGen Precision Health, University of Missouri, 416 South 6th Street, Columbia, MO 65211, United States; Department of Electrical Engineering and Computer Science, NextGen Precision Health, University of Missouri, 416 South 6th Street, Columbia, MO 65211, United States; Department of Electrical Engineering and Computer Science, NextGen Precision Health, University of Missouri, 416 South 6th Street, Columbia, MO 65211, United States; Bond Life Sciences Center, University of Missouri, 1201 Rollins Street, Columbia, MO 65211, United States; Health Informatics Institute, Morsani College of Medicine, University of South Florida, 3650 Spectrum Boulevard, Tampa, FL 33612, United States

**Keywords:** Cryo-EM, protein particle picking, segment anything model 2 (SAM2), parameter-efficient adapter, few-shot learning, image segmentation

## Abstract

Accurate identification of protein particles in cryo-electron microscopy (cryo-EM) micrographs is crucial for high-resolution structure determination, but remains challenging due to the heavy reliance on extensive annotated datasets and the difficulty of ensuring robustness under low signal-to-noise ratio (SNR) conditions. Current approaches require large annotations and exhibit poor generalization to new protein targets. We present CryoFSL (Cryo-EM Few Shot-Learning), a novel few-shot learning framework built on Segment Anything Model 2 with lightweight adapters, enabling robust particle picking with as few as five labeled micrographs and significantly reducing the annotation burden. The framework’s hierarchical adapter design supports dynamic feature modulation for low-SNR and heterogeneous conditions, resolving the trade-off between annotation burden and performance. CryoFSL surpasses both traditional template-based methods and state-of-the-art deep learning models across diverse proteins in the few-shot learning setting, achieving superior recall, precision, and 3D reconstruction resolution with minimal supervision. It maintains stability across heterogeneous micrographs and consistently detects high-quality particles with fewer false-positives. Notably, CryoFSL achieves competitive resolution in density map reconstruction with just a fraction of the particles picked by other methods, redefining efficiency and quality in cryo-EM analysis. This work paves the way for scalable, generalizable, and annotation-efficient particle-picking pipelines. The code is available at https://github.com/biplabpoudel25/CryoFSL.

## Introduction

The determination of protein structures stands at the forefront of modern structural biology, providing essential insights into cellular mechanisms, disease processes, and therapeutic development [1]. Understanding the structure of proteins is essential for elucidating molecular function, characterizing biomolecular interactions, interpreting disease-associated alterations, and advancing drug development [2, 3]. Cryo-electron microscopy, or cryo-EM, has emerged as a groundbreaking technology for structure determination, enabling near-atomic resolution imaging of large macromolecular complexes by preserving specimens in their native state through rapid vitrification [4–6].

A pivotal step in the cryo-EM workflow is protein particle picking, which involves identifying and extracting individual protein particles from micrographs containing thousands of randomly oriented molecules in noisy backgrounds [7]. This process presents substantial technical challenges that directly influence the quality of downstream structural analysis. Cryo-EM micrographs are inherently characterized by extremely low signal-to-noise ratios (SNR), rendering protein particles as subtle contrast variations that are often indistinguishable from background noise, ice contamination, and various imaging artifacts [8, 9]. The complexity is further compounded by the intrinsic heterogeneity of biological specimens, which may exhibit conformational flexibility, preferred orientations, and structural variability. Additionally, aggregated particles, overlapping structures, and false-positives (FPs) such as ice crystals further complicate reliable particle detection [10–12]. Therefore, robust and efficient particle picking is essential for ensuring high-resolution cryo-EM structures, as the accuracy and effectiveness of this process have a significant impact on the quality of the resulting 3D density map reconstruction and its resolution.

Traditional particle-picking approaches have progressed from fully manual selection to semi-automated template-based methods incorporated in widely used software packages such as EMAN2 [13], RELION [14], Scipion [15], Dog Picker [16], and APPION [17]. Manual picking, while accurate, is labor-intensive and unsuitable for large datasets [18, 19]. Template-based picking methods involve the generation of 2D reference templates from initial particle subsets, which are then cross-correlated with the entire micrograph through iterative refinement to guide the particle identification [18]. Although these approaches have demonstrated effectiveness for well-characterized proteins under favorable signal-to-noise conditions, their performance remains constrained by the quality and representativeness of the templates. The inherent limitations of template-based methods include their dependence on iterative user intervention, extensive parameter optimization requirements, and susceptibility to template selection bias. These constraints limit their applicability when applied to new targets or heterogeneous datasets and introduce operator-dependent variability that can compromise reproducibility. Furthermore, templates may inadequately represent variations in particle orientation, size, or imaging conditions, leading to reduced accuracy in complex experimental scenarios [18, 20–23].

Advancements in deep learning methods have shown great promise for particle-picking automation strategies. A number of models, such as APPLE picker [23], DeepPicker [24], AutoCryoPicker [25], Warp [26], CASSPER [27], Topaz [28], CrYOLO [29], and CryoMAE [30] have been developed to improve detection accuracy and reduce manual intervention. Among them, Topaz and CrYOLO, both adopting convolutional neural network-based models, remain the most widely used in the cryo-EM community. However, CrYOLO frequently overlooks true protein particles in micrographs, whereas Topaz is susceptible to FP detection, including ice contaminants and duplicate particles [30–32]. More recent deep learning approaches, including CryoSegNet [31] and CryoTransformer [32], have achieved improved performance through sophisticated architectural designs and extensive training. However, these methods typically require large, well-curated training datasets and substantial computational resources for model optimization, making it challenging to apply them in scenarios where limited training data are available [21]. The reliance on extensive training data also raises concerns about generalization to novel particle types or experimental conditions not well-represented in the training datasets. The trade-offs between annotation burden and model adaptability underscore a key gap in cryo-EM, a paradigm that balances efficient learning with strong flexibility under extreme data scarcity.

Recent advances in biomedical image segmentation have highlighted the value of lightweight, hierarchical architectures that balance computational efficiency and multi-scale representation learning. Foundational and large-scale vision models such as Segment Anything Model 2 (SAM2) [33] have been successfully adapted for diverse biomedical-segmentation tasks, including 3D tumor segmentation in breast ultrasound [34], polyp and surgical tool detection in endoscopy [35], and general medical image segmentation [36, 37], through parameter-efficient fine-tuning and hierarchical feature refinement strategies. These developments motivate the adoption of similar principles for cryo-EM particle picking, where annotation scarcity and imaging complexity demand efficient solutions. This study presents CryoFSL (Cryo-EM Few Shot-Learning), a data-efficient, few-shot learning framework for protein particle picking in cryo-EM micrographs, leveraging the SAM2, a state-of-the-art vision foundation model. Our approach incorporates lightweight adapter modules into SAM2’s image encoder while keeping the base model frozen, enabling rapid and effective adaptation to novel protein specimens with as few as five manually annotated micrographs. Although built on a deep learning foundation, CryoFSL’s working mechanism closely resembles template-based methods in practice, offering efficient adaptation to new targets with minimal supervision and no need for large-scale retraining. CryoFSL is specifically designed for the practical settings where current particle-picking methods struggle: (i) novel or low-resource projects where only a handful of annotated micrographs are available (e.g. early screening of a new target or small labs without extensive annotation resources); (ii) low signal-to-noise and heterogeneous datasets where particle contrast varies strongly across micrographs and templates or fully pretrained supervised models fail to generalize; (iii) workflows that prioritize particle quality over raw quantity, such as downstream projects requiring high-quality reconstruction from fewer and cleaner particles; and (iv) computationally constrained environments where full model retraining is impractical. By directly addressing these challenges, CryoFSL bridges the gap between accuracy, annotation efficiency, and generalizability, providing a practical solution for both exploratory and large-scale cryo-EM studies.

We tested CryoFSL against traditional template-based (EMAN2, RELION, Scipion) and deep learning methods (Topaz, CrYOLO), demonstrating superior performance across diverse proteins. Models like CryoSegNet and CryoTransformer, while effective in fully supervised settings, were excluded due to their architectural design for full fine-tuning, which lacks native support for parameter-efficient adaptation required for robust few-shot learning. Consequently, evaluating them under identical few-shot conditions (five micrographs) would require architectural modifications beyond their current public releases, making direct comparison methodologically inconsistent with our focus on annotation-efficient adaptations. Therefore, we benchmarked against methods with established pipelines for low-data training to ensure a fair evaluation of annotation efficiency. Within this defined comparison, CryoFSL excels in low SNR and high-heterogeneity scenarios, demonstrating the promise of integrating foundational models with few-shot learning to deliver scalable, accurate, and annotation-efficient particle picking for structural biology.

## Materials and methods

### Dataset

We evaluate our approach using the CryoPPP dataset, a large and diverse collection of expertly annotated cryo-EM micrographs curated from the EMPIAR. CryoPPP encompasses a wide range of protein types, molecular sizes, particle shapes, and imaging conditions, including low SNR, ice contamination, carbon films, and heterogeneous particle distributions. For our few-shot experiments, we select a representative subset of six proteins from CryoPPP (EMPIARs-10028, 10081, 10017, 10093, 10345, 11056). These datasets were chosen to reflect variability in particle morphology and micrograph complexity. For each protein, we randomly sample 1, 5, and 10 labeled micrographs to simulate 1-, 5-, and 10-shot scenarios, respectively. The details of the dataset are presented in Supplementary Table S1.

### Evaluation of particle picking

To quantitatively assess the particle-picking performance of CryoFSL and competing approaches, we adopted four widely used metrics: precision, recall, F1 score, and IoU. Precision measures the proportion of correctly identified particles among all predicted particles, whereas recall measures the proportion of GT particles that were correctly detected.


$$Precision=\frac{TP}{TP+ FP}$$

$$Recall=\frac{TP}{TP+ FN}$$

Here, true positive (*TP*) denotes the predicted particles correctly matched to GT, *FP* denotes the predicted particles with no matching GT, and false negative (*FN*) denotes the GT particles that were missed.

F1 score is the harmonic mean of precision and recall, providing a balanced metric for performance.


$$F1- score=\frac{2\times Precision\times Recall}{Precision+ Recall}$$

IoU is a measure of the spatial overlap between predicted and GT particles.


$$IoU=\frac{area\ \left({P}_D\cap{P}_T\right)}{area\ \left({P}_D\cup{P}_T\right)}$$

where ${P}_D$ represents the predicted particles, and ${P}_T$ represents the GT particles.

To further validate the impact of particle-picking accuracy on downstream structure determination, we also report the 3D reconstruction resolution obtained using particles picked by each method. This metric reflects the quality of structural recovery and is reported in Angstroms (Å), where lower values indicate higher quality.

### Statistical analysis

To evaluate the statistical significance of differences between CryoFSL and competing methods, paired Wilcoxon signed-rank tests were applied per micrograph across the six EMPIAR datasets. Prior to testing, Shapiro–Wilk tests [38] confirmed that 95.8% of per-micrograph metric distributions significantly violated normality (*P*-value <0.05). This is expected given the inherently heterogeneous nature of cryo-EM micrographs, where variable particle density, differing SNRs, and imaging artifacts collectively produce highly skewed and non-Gaussian metric distributions across micrographs. Given this widespread violation of parametric assumptions, the Wilcoxon signed-rank test was employed as a robust nonparametric paired test to compare paired metric values (precision or recall), testing the null hypothesis of no difference against CryoFSL as the reference method. Effect sizes were calculated to quantify the magnitude and direction of differences using the rank-biserial correlation [39], computed as $r=z/\sqrt{N}$, where $z$ is the Wilcoxon test statistic and $N$is the number of paired observations.

Given multiple comparisons (5 competitor methods × 6 datasets = 30 tests per metric), raw *P*-values were adjusted for false discovery using the Benjamini–Hochberg procedure [40] for controlling the false discovery rate. All statistical computations were implemented in Python using the $scipy. stats. wilcoxon$ function for raw *P*-values and effect sizes, and $statsmodels. stats. multitest. multipletests$ with the “$fdr\_ bh$” method for adjustments.

### Overall framework

Our framework leverages the SAM2 as the backbone for automated protein particle picking in cryo-EM micrographs. SAM2 features a Hiera-large hierarchical vision transformer encoder, which efficiently captures multi-scale visual representations through progressive down-sampling and deepening of features. This hierarchical structure enables the model to integrate both local texture and global context, which are crucial for detecting protein particles in noisy, low-contrast micrographs.

The proposed architecture consists of four main components: the frozen SAM2 Hiera-large image encoder for robust feature extraction, novel lightweight adapter modules integrated across the encoder’s stages for task-specific adaptation, the SAM2 mask decoder for segmentation, and a postprocessing pipeline to extract particle coordinates from the segmentation mask. Unlike the original SAM [41]/SAM2, CryoFSL omits the prompt encoder and operates without any interactive inputs (e.g. points, boxes, or masks), enabling fully automated particle detection.

### Adapter module design

To adapt the frozen SAM2 image encoder for the particle-picking task, we used parameter-efficient adapter modules following the approaches of Chen *et al*. [42] and He *et al*. [43], strategically placing them across all four hierarchical stages of the encoder. In our implementation, the adapter is applied as a residual feature modulation before each transformer block, i.e. a learned adaptation is computed and added to the block input feature map. Each adapter operates on two input streams: a high-pass Fast Fourier Transform (FFT)-filtered representation of the input micrograph, capturing frequency-domain structural cues, and a linearly projected version of the current block’s feature embeddings, capturing stage-specific semantic context. These features are projected to a reduced dimension (1/32 of the stage embedding size), summed, and passed through a block-specific unshared linear layer $\left({L}_{unshared}\right)$, followed by Gaussian Error Linear Unit (GELU) activation, enabling block-level specialization. The resulting feature is then projected back to the full embedding dimension by a shared linear layer (${L}_{shared}$) common to all blocks within the same stage, ensuring feature dimensionality. The final adapter output is added to the transformer block input via a residual connection, ensuring task-specific modulation without disrupting the pretrained encoder’s representations.

The varying capacities of adapters ${A}_1$ through ${A}_4$ reflect this hierarchical progression: adapter ${A}_1$ operates on the highest spatial resolution features (Stage 1, 144-dimensional embeddings) with two lightweight adapters. At Stage 2, adapter ${A}_2$ processes 288-dimensional embeddings and six adapters. At Stage 3, adapter ${A}_3$ handles 576-dimensional embeddings, comprising 36 adapters, the largest capacity among the stages, consistent with the deeper representation complexity. Finally, adapter ${A}_4$ is integrated into Stage 4, where 1152-dimensional embeddings are projected with four adapters. This architectural design ensures that each hierarchical level benefits from appropriately scaled adaptation capacity, balancing spatial detail in the early stages with semantic richness in the deeper layers.

Let $i\in \left\{1,2,3,4\right\}$ denote the stage index, and $j\in \{0,1,\dots, depths[i-1]\}$ represent the block index within that stage. Let ${feat}_{\left(i,j\right)}$ be the input feature to block $j$ in stage $i$. Each adapter consists of an unshared linear layer ${L}_{\left(i,j\right)}^{unshared}$ specific to block, GELU activation function $\sigma$, and a shared linear layer ${L}^{shared}$ that is common across all adapters. The adapter output for block $j$ in stage $i$ is formulated as:


$${feat}_{\left(i,j\right)}^{adapter}={L}^{shared}\ \left(\sigma\ \left({L}_{\left(i,j\right)}^{unshared}\ \left({feat}_{\left(i,j\right)}\right)\right)\right)$$

Or, in simplified notation:


$${feat}_{\left(i,j\right)}^{adapter}={W}^{shared}.\sigma\ \left({W}_{\left(i,j\right)}^{unshared}.\kern0.5em {feat}_{\left(i,j\right)}\right)$$

where ${W}_{\left(i,j\right)}^{unshared}$ and ${W}^{shared}$are the learnable weight matrices of the unshared and shared layers, respectively. The task-adapted feature is then added to the original input feature via a residual connection and the final output of block $j$ in stage $i$ is:


$${feat}_{\left(i,j\right)}^{final}={feat}_{\left(i,j\right)}+{feat}_{\left(i,j\right)}^{adapter}$$

This residual formulation ensures the adapter introduces task-specific modulation without disrupting the pretrained encoder’s underlying representations. Finally, the updated feature ${feat}_{\left(i,j\right)}^{final}$ is forwarded through the transformer block ${BLK}_{\left(i,j\right)}$ for further processing as:


$${feat}_{\left(i,j+1\right)}={BLK}_{\left(i,j\right)}\ \left({feat}_{\left(i,j\right)}^{final}\right)$$

This integration strategy allows each block in the frozen SAM2 encoder to benefit from targeted, low-parameter task adaptation, enabling the model to generalize effectively from a limited number of labeled examples without full fine-tuning of the encoder weights.

### Postprocessing and particle localization

To extract accurate particle coordinates from SAM2-generated masks, we employ a robust multi-stage postprocessing pipeline combining distance transform, multi-scale peak detection, and watershed segmentation. Geometric filtering based on circularity and area constraints ensures biological plausibility, while a dual-pass strategy enables recovery of closely packed or overlapping particles without duplication. A schematic overview and a detailed algorithm are provided in Supplementary Fig. S1 and Supplementary Algorithm S1, respectively.

### Training

CryoFSL was trained on five annotated micrographs from each EMPIAR dataset. All micrographs were resized to 1024 × 1024 pixels to align with the input requirements of the SAM2 model. Training was performed using a batch size of two, the Adam optimizer [44] with a learning rate of $1\times{10}^{-4}$ and a maximum of 4000 epochs. Although the maximum was set to 4000 epochs, the model consistently converged earlier in practice, typically between 1500 and 3000 epochs depending on dataset complexity. Proteins with higher particle density and clearer structural features (e.g. EMPIAR-10028) converged faster, while more challenging datasets with lower SNR (e.g. EMPIAR-10345) required more epochs. The 4000-epoch limit was chosen to ensure full convergence across all datasets. The total number of trainable parameters in the model was ~3.94 million. For optimization, the balanced binary cross-entropy loss with logits (BCEWithLogitsLoss) was used. During training, the SAM2 image encoder was frozen, and only the adapter module and SAM2 mask decoder were updated. All experiments were implemented in Python 3.11.0 using PyTorch 2.3.0 with CUDA 11.8 acceleration. Computations were performed on a Linux-based cluster equipped with NVIDIA A100 80GB GPUs, enabling efficient training and testing.

Template-based methods (EMAN2, RELION, and Scipion) were evaluated using their standard correlation-based workflows. For each protein, templates were generated from the same five labeled micrographs and applied to the test sets with default settings, adjusting only diameter and correlation thresholds when required. No additional fine-tuning or external data were used. Deep learning approaches were likewise trained under identical few-shot configurations. For CrYOLO, we employed the PhosaurusNet architecture with an input image of size 768 × 768 and trained for 100 epochs with a learning rate of $1\times{10}^{-4}$. Similarly, Topaz used a ResNet backbone, configured with 32 units in the initial layers and batch normalization, trained for 50 epochs with a learning rate of $2\times{10}^{-4}$ using the Generalized Expectation (GE)-binomial loss. All approaches were then applied to the same test datasets, ensuring a direct and fair comparison with CryoFSL under sparse annotation regimes.

## Results

### CryoFSL framework for few-shot protein particle picking

**Figure 1:**
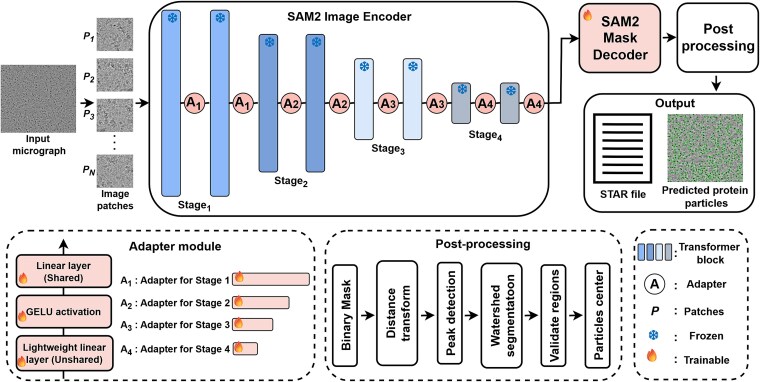
CryoFSL architecture for few-shot cryo-EM protein particle picking; a small set of annotated micrographs is processed by the SAM2 image encoder, augmented with lightweight adapter modules for efficient fine-tuning under limited supervision; the encoder comprises four sequential stages (${Stage}_1$to ${Stage}_4$), each with transformer blocks (vertical bars), and adapters (${A}_1$–${A}_4$) inserted between respective blocks; frozen and trainable components are visually distinguished to emphasize CryoFSL’s minimal parameter footprint; encoded features are decoded into binary masks, followed by structured postprocessing to extract particle coordinates; final outputs include a STAR file and visual overlays of predicted particles.

We introduce CryoFSL, a parameter-efficient few-shot learning framework for automated protein particle picking in cryo-EM micrographs, utilizing SAM2 enhanced with task-specific adapter modules. As illustrated in Fig. 1, input micrographs are encoded using the frozen SAM2 Hiera-large encoder to extract multiscale features, which are crucial for detecting particles in noisy, low-contrast images. To enable task-specific adaptation without full finetuning, we insert adapters at each encoder stage (see Methods). Adapted features are decoded by the SAM2 mask decoder into segmentation masks, which are postprocessed to extract precise particle coordinates (Supplementary Algorithm S1). Final outputs include STAR-formatted coordinates compatible with CryoSPARC [45] and visual overlays of detected particles.

In practical cryo-EM context, “few-shot” refers to the number of annotated micrographs rather than individual particle instances, as micrograph inspection represents the primary annotation bottleneck due to high particle density per image. We conducted extensive experiments on the CryoPPP dataset [46] across 1-, 5-, and 10-shot scenarios on six Electron Microscopy Public Image Archive (EMPIAR) proteins (10028, 10081, 10017, 10093, 10345, 11056) [47], using precision, recall, F1 score, Intersection over Union (IoU), and 3D reconstruction resolution as metrics. All baseline methods were trained on five annotated micrographs per protein to ensure a fair comparison. Each method was configured according to published best practices (see Methods for details). CryoFSL consistently outperformed all baselines, demonstrating superior stability and accuracy in low-annotation, few-shot settings.

### Comparative evaluation of 1-, 5-, and 10-shot learning for CryoFSL particle picking

**Figure 2:**
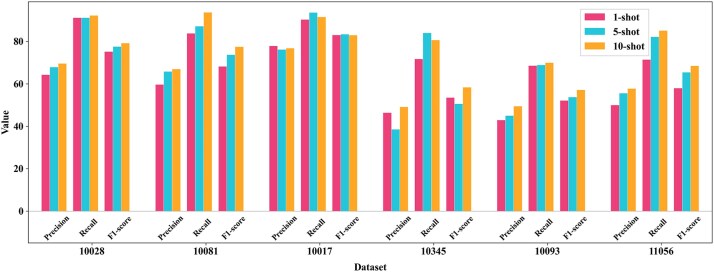
Detailed comparison of CryoFSL’s performance across 1-, 5-, and 10-shot learning scenarios, evaluated using precision, recall, and F1 score metrics on six diverse cryo-EM protein datasets from the EMPIARs (10028, 10081, 10017, 10345, 10093, and 11056); the *x*-axis shows the six datasets, and the *y*-axis displays the numerical values of the performance metrics, ranging from 0 to 100, with higher values indicating better performance; three few-shot learning scenarios are compared using color-coded bars: 1-shot learning (pink), 5-shot learning (cyan), and 10-shot learning (orange), where each scenario corresponds to the number of annotated micrographs used for training.

We assessed CryoFSL’s few-shot learning capabilities under 1-, 5-, and 10-shot configurations, where the model was trained using 1, 5, and 10 annotated micrographs per protein, respectively, to investigate the trade-off between annotation effort and model performance. The results are shown in Fig. 2. As expected, the 10-shot setup achieved the highest performance, particularly excelling in datasets like 10081, 10093, and 11056. Impressively, the 1-shot scenario revealed CryoFSL’s tolerance for sparse annotations by achieving an acceptable F1 score above 50% on most proteins despite only using one annotated micrograph.

The progression from 1-shot to 5-shot yielded greatly enhanced performance, with the 5-shot configuration achieving results remarkably close to 10-shot across all datasets. In certain instances, such as EMPIARs-10017 and 10345, the 5-shot CryoFSL outperformed 10-shot learning, achieving higher recall scores. Notably, the marginal gains from 5-shot to 10-shot settings were modest, with 5-shot achieving 90%–95% of the maximum attainable performance while requiring only half the annotated data and computational resources. Consequently, we adopted the 5-shot configuration as our optimal training paradigm for all subsequent experiments, as it struck an ideal balance between performance excellence and practical feasibility. This choice supported one of the central goals of our framework: achieving strong generalization with minimal supervision, thereby enabling rapid and scalable deployment in real-world cryo-EM analysis workflows.

### Systematic comparison of methods using segmentation metrics across diverse cryo-EM datasets in few-shot learning settings

CryoFSL demonstrated excellent overall performance across all six protein targets, achieving the highest average recall (0.845) and F1 score (0.684), as shown in Table 1. In contrast, template-based methods like RELION, EMAN2, and Scipion achieved high recall (e.g. 0.793 for RELION and 0.787 for Scipion) but considerably lower precision (0.352 and 0.367), resulting in suboptimal F1 scores of 0.471 and 0.478. Even deep learning methods designed for low-data regimes, such as Topaz and CrYOLO, struggled in challenging cases like EMPIAR-10345, where they achieved F1 scores of just 0.074 and 0.078, respectively. Supplementary Fig. S2 confirms their failure to identify many true particles, reflecting poor adaptability under morphological complexity and low contrast. This suggests that such methods may require more labeled data to achieve reliable results. In contrast, CryoFSL achieved a stable F1 score of 0.528 on the same dataset, leveraging adaptive feature modulation to effectively capture particle characteristics. This resilience with minimal labeled data highlights CryoFSL’s efficiency in few-shot settings, significantly reducing the need for extensive annotations and enhancing reliability for complex cryo-EM micrographs.

**Table 1:** Quantitative evaluation results on six protein datasets from CryoPPP for a few-shot setting.

EMPIAR IDs	Protein type	Metrics	CrYOLO	Topaz	EMAN2	RELION	Scipion	CryoFSL
10028 [48]	Ribosome (80S)	Precision	**0.741**	0.693	0.444	0.501	0.448	0.678
		Recall	0.764	0.749	0.875	0.899	0.898	**0.911**
		F1 score	0.752	0.719	0.589	0.643	0.597	**0.777**
		IoU	0.602	0.562	0.417	0.474	0.426	**0.636**
10081 [49]	Transport	Precision	0.641	0.590	0.353	0.377	0.365	**0.658**
		Recall	0.665	0.850	0.805	0.804	0.807	**0.872**
		F1 score	0.652	0.696	0.491	0.513	0.502	**0.751**
		IoU	0.484	0.534	0.325	0.345	0.335	**0.600**
10017 [50]	$\beta$ -Galactosidase	Precision	0.754	0.742	0.591	0.372	0.500	**0.762**
		Recall	0.361	0.709	0.552	0.753	0.832	**0.936**
		F1 score	0.488	0.725	0.571	0.497	0.624	**0.839**
		IoU	0.322	0.567	0.399	0.331	0.454	**0.724**
10345 [51]	Signaling	Precision	0.142	0.047	0.133	0.105	0.080	**0.386**
		Recall	0.054	0.185	0.623	**0.874**	0.858	0.840
		F1 score	0.078	0.074	0.219	0.187	0.143	**0.528**
		IoU	0.041	0.039	0.123	0.103	0.078	**0.359**
10093 [52]	Membrane	Precision	0.536	**0.571**	0.376	0.385	0.324	0.450
		Recall	0.478	0.434	0.511	0.628	0.596	**0.689**
		F1 score	0.505	0.493	0.433	0.477	0.419	**0.544**
		IoU	0.338	0.327	0.276	0.314	0.266	**0.373**
11056 [53]	Transport	Precision	**0.670**	0.624	0.507	0.372	0.482	0.556
		Recall	0.496	0.478	0.608	0.799	0.734	**0.822**
		F1 score	0.571	0.541	0.553	0.507	0.581	**0.663**
		IoU	0.399	0.371	0.382	0.340	0.409	**0.495**
Average		Precision	0.581	0.545	0.401	0.352	0.367	**0.582**
		Recall	0.470	0.568	0.662	0.793	0.787	**0.845**
		F1 score	0.508	0.541	0.476	0.471	0.478	**0.684**
		IoU	0.364	0.400	0.320	0.318	0.328	**0.531**

Column 1 lists the EMPIAR IDs, and column 2 specifies the corresponding protein types. The remaining column reports the evaluation metrics (precision, recall, F1 score, and IoU) for each compared method. For each metric, the best-performing method is highlighted in bold. The final row presents the average performance of each method across all six proteins, with the best average also highlighted.

**Figure 3:**
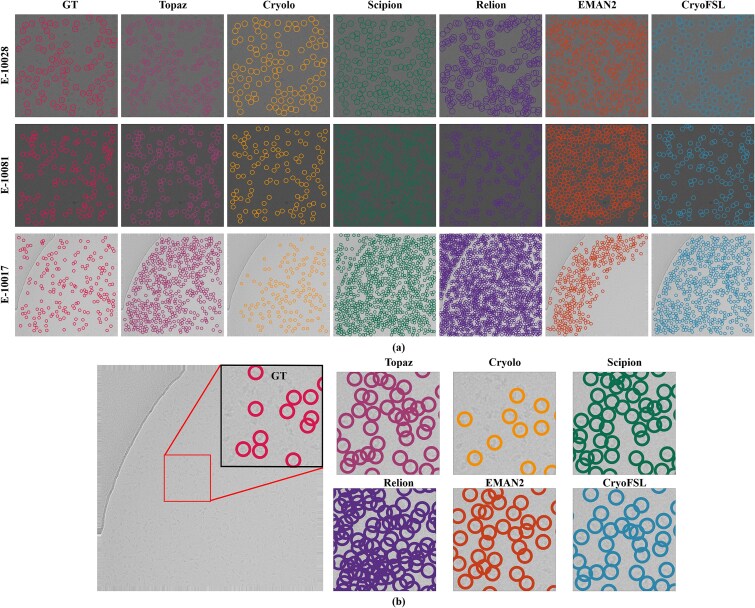
Visual comparison of particle picking across methods and sample micrographs; (a) sample micrographs from EMPIAR IDs 10028, 10081, and 10017 are shown with particle locations predicted by all competing methods, overlaid alongside the expert-annotated GT; each method’s result is shown as colored circular markers on the micrographs; all models were evaluated on the same set of micrographs to ensure consistency in qualitative comparison; (b) a magnified view of a representative region from a micrograph in EMPIAR-10017 highlights differences in particle localization between the methods; the red box on the full micrograph shows the zoomed region.

Visual comparisons of selected micrographs from EMPIARs-10028, 10081, and 10017 further illustrate these differences (Fig. 3). CrYOLO generally under-picks, missing a large number of true particles. Topaz, while capturing most particles from the ground truth (GT), suffers from high redundancy due to frequent overlapping picks. On the other hand, template-based methods like RELION, Scipion, and EMAN2 exhibit aggressive over-picking behavior, identifying excessive numbers of FPs and including non-particle regions, leading to noisy and less reliable selections. This is especially evident in the magnified regions of Fig. 3b, where multiple overlapping or ambiguous picks are observed. CryoFSL, however, achieves a strong balance: maintaining precise alignment with expert-annotated GT particles while simultaneously detecting additional valid particles overlooked during manual curation. Supplementary Figs S3–S7 visually reinforce this, highlighting the widespread false detections by competing methods and the clean, uniform particle selections achieved by CryoFSL.

### Robustness and failure mode analysis of particle-picking methods using segmentation metric distributions

**Figure 4:**
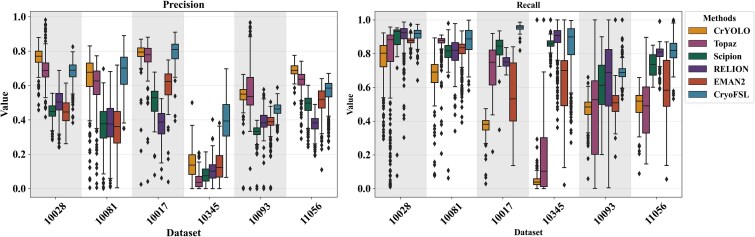
Detailed box plots comparing precision (left) and recall (right) distribution across six methods on six different datasets; the *x*-axis for both plots represents the six datasets (10028, 10081, 10017, 10345, 10093, and 11056), while the *y*-axis displays the metric values, ranging from 0 to 1; each box represents the interquartile range, the black horizontal line inside each box indicates the median value, and the whiskers extend to the range of non-outlier values; outliers are plotted as individual points.

The boxplot analysis in Fig. 4 reveals considerable variability in precision and recall distributions across methods, as evidenced by wider interquartile ranges and frequent outliers, particularly for template-based approaches such as RELION and EMAN2. These large spreads and skewed distributions indicate unstable particle-picking behavior across different micrographs, often due to over-picking in cluttered or noisy regions. The outliers represent instances in which template correlation thresholds are either too permissive (resulting in excessive FPs) or too restrictive (missing true targets), suggesting that fixed-parameter approaches cannot dynamically adapt to the heterogeneous nature of cryo-EM micrographs. This phenomenon is especially pronounced in EMPIARs-10345 and 10093, where particle diversity and contamination challenge the reproducibility of the templates. The resulting inconsistency is also reflected in Table 1, where high recall is often offset by low precision and F1 score.

In contrast, CryoFSL achieves comparatively tighter distributions across both precision and recall metrics, with fewer outliers and generally higher medians than other methods across most protein datasets. While variability persists in some cases, the narrower interquartile ranges indicate more stable behavior that adapts dynamically to varying imaging conditions rather than relying on static decision boundaries. This stability stems from CryoFSL’s adapter modules’ ability to modulate feature representation in response to local micrograph characteristics, effectively learning to distinguish signal from noise patterns that would confound correlation-based approaches. Statistical analysis using Wilcoxon signed-rank tests [54] confirms CryoFSL’s superior performance, with highly significant adjusted *P*-values (e.g. <$1\times{10}^{-10}$for recall in most cases) and large effect sizes, as detailed in Supplementary Tables S2 and S3. Further, threshold-based analysis shows that CryoFSL achieves 96.6% recall at the 0.6 threshold and maintains 69.1% at 0.8, outperforming all baselines in both performance and consistency, as detailed in Supplementary Fig. S8.

A combined analysis of segmentation behavior across multiple EMPIAR-10345 micrographs (Supplementary Fig. S7 and Supplementary Table S4) highlights distinct failure modes among methods. CryoFSL maintains particle predictions within a stable range (typically 100–150), reflecting high recall and robustness, with acceptable precision due to the detection of valid but unlabeled particles. In contrast, CrYOLO consistently under-picks (as few as 9–26), while Topaz exhibits highly variable behavior, with predicted counts ranging from 5 to 363 depending on micrograph complexity. Template-based methods such as Scipion, RELION, and EMAN2 exhibit aggressive over-picking, achieving high recall but extremely low precision due to genuine FP detections. Notably, CryoFSL’s lower precision arises from the recovery of true but unlabeled particles, fundamentally different from the FP-driven precision loss in traditional methods.

### Comparative analysis of particle quantity and 3D density map reconstruction resolution across methods

**Figure 5:**
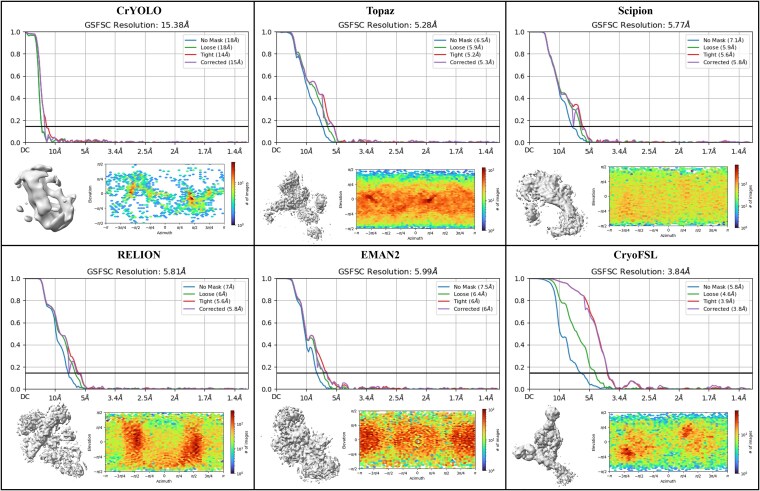
Comparison results for the resolution of the 3D density maps and reconstructed 3D density maps of particles picked by different methods on the EMPIAR-10345 dataset.

Table 2 presents a comprehensive evaluation of particle quantity and the quality of 3D density map reconstruction across methods. Template-based methods routinely selected two to three times more particles than our approach, yet CryoFSL achieved the best average resolution of 5.33 Angstroms (Å) with significantly fewer particles—around 45 000 on average. This trend is particularly striking for challenging proteins like 10345, where CryoFSL’s selection of 21 008 particles yielded exceptional 3.84 Å resolution, outperforming all other methods by greater than 1.44 Å despite their much larger particle sets. It is important to note that the relationship between particle count and resolution is complex and is influenced by factors such as orientation distribution and conformational heterogeneity. Nevertheless, methods picking substantially more particles consistently yielded larger resolution values—for instance, RELION reached only 7.36 Å on EMPIAR-10028 with 68 055 particles, and EMAN2 yielded 7.26 Å on EMPIAR-10081 with 97 234 particles. This suggests that indiscriminate picking may introduce particles less amenable to high-resolution reconstruction under these experimental conditions. These observations are consistent with findings from Table 1 and Fig. 3, where traditional methods exhibited high recall but low precision and significant performance variability. The 3D density map visualizations for EMPIAR-10345 are shown in Fig. 5, showing clearer structural features using CryoFSL, with additional reconstructions in Supplementary Figs S9 and S10.

**Table 2:** Comparison of 3D reconstruction resolution and particle yield across six cryo-EM protein datasets.

EMPIAR IDs	CrYOLO		Topaz		EMAN2		RELION		Scipion		CryoFSL	
	#	Res.	#	Res.	#	Res.	#	Res.	#	Res.	#	Res.
10028	26121	4.15	36112	**4.08**	59582	4.25	68055	7.36	56591	4.13	30043	4.13
10081	36474	7.21	56753	6.51	97234	7.26	104452	8.00	85168	7.36	31799	**6.16**
10017	20793	5.98	47462	5.43	48132	5.61	98263	5.58	84108	5.89	45349	**4.99**
10345	4256	15.38	66247	5.28	83375	5.99	118637	5.81	125693	5.77	21008	**3.84**
10093	42983	6.72	55636	6.80	77593	7.34	101355	7.11	86495	6.72	37513	**6.47**
11056	83713	8.09	101842	7.11	183448	7.64	157005	7.40	188497	6.59	106643	**6.44**
Average	35723	7.92	60675	5.86	91560	6.34	107961	6.87	104425	6.07	45392	**5.33**

For each EMPIAR IDs, the number of particles picked (#) and the corresponding 3D resolution (res., in Å) obtained using each method are reported. The final row shows the average particle count and average resolution for each method. The best resolution (lowest res. Value) for each protein is highlighted in bold, indicating superior performance in structural recovery.

**Figure 6:**
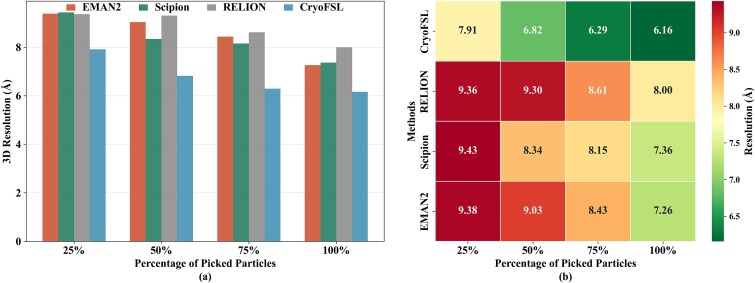
3D reconstruction resolution analysis at different particle sampling levels for EMPIAR-10081; (a) bar plot: resolution (Å) achieved by each method using 25%, 50%, 75%, and 100% of their picked particles; the *x*-axis shows sampling percentage, and the *y*-axis shows resulting resolution, with lower bars indicating better performance; (b) heatmap: same data visualized with color-coded resolution values; rows represent methods, columns indicate sampling levels, and cell colors range from green (better resolution) to red (worse resolution).

To further examine reconstruction behavior across particle subsets, we conducted a progressive reconstruction analysis on EMPIAR-10081 protein, evaluating resolution at 25%, 50%, 75%, and 100% of the particles picked. Figure 6 reveals that with just 25% of the particles, CryoFSL achieves a resolution of 7.91 Å, already comparable to or better than the full 100% sets of EMAN2 (7.26 Å), Scipion (7.36 Å), and RELION (8 Å). As more particles are added, resolution for CryoFSL improves steadily—6.82 Å at 50%, 6.29 Å at 75%, and 6.16 Å at 100%, but the diminished gain beyond 50% reflects the robust and high-quality particles across its selection. In contrast, template-based methods like EMAN2 and Scipion show larger performance jumps (e.g. EMAN2 improves from 9.38 Å at 25% to 7.26 Å at 100%). This steeper improvement may reflect a higher proportion of difficult-to-align or noisy particles in their selections, though differences in orientation coverage and heterogeneity could also contribute to this pattern.

### Evaluating the impact of annotation density on 3D resolution performance across picking methods

**Figure 7:**
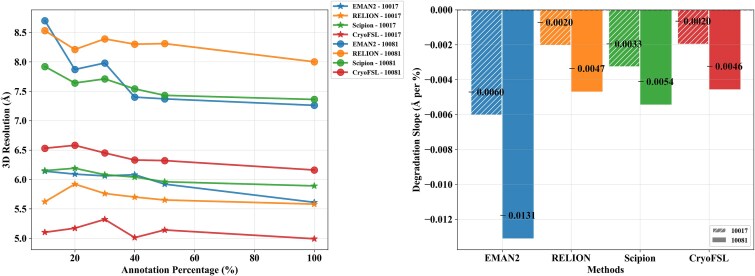
Robustness analysis of particle-picking methods across varying annotation levels on EMPIARs-10017 and 10081; (a) 3D resolution (Å) achieved by each method at different annotation percentages; the *x*-axis shows training annotation percentage, and *y*-axis shows resolution (lower is better); markers indicate proteins: ★ for 10017 and ● for 10081; flatter curves reflect higher robustness, and steeper declines indicate greater sensitivity to reduced annotations; (b) degradation slope (Å per %), measuring resolution loss per 1% decrease in annotation; the *x*-axis lists methods and the *y*-axis shows the degradation slope; lower magnitudes (closer to 0) indicate better robustness; a dashed line at *y* = 0 indicates ideal robustness (no degradation).

To examine performance under limited supervision, we trained each method on five micrographs per protein while varying particle annotations from 10% to 100%. As shown in Fig. 7a and Supplementary Table S5, CryoFSL demonstrates stable resolution across annotation levels, with only marginal gains at higher supervision. For example, on protein 10017, CryoFSL achieves 5.10 Å at 10% annotation, nearly matching its 4.99 Å at 100%, and still outperforms EMAN2 (5.61 Å), Scipion (5.89 Å), and RELION (5.58 Å) trained with full annotations. A similar trend holds for protein 10081. This robustness is further quantified in Fig. 7b, where CryoFSL shows the flattest resolution degradation slopes (e.g. −0.00197 Å/% for 10017), indicating minimal sensitivity to annotation sparsity. In contrast, traditional methods degrade sharply as the number of annotations decreases. These findings highlight CryoFSL’s robustness under sparse supervision and its ability to maintain reconstruction performance with limited annotations.

## Discussion

Cryo-EM protein particle picking is a crucial yet challenging step in structural biology due to extremely low SNR, subtle contrast variations, and imaging artifacts such as ice contamination. Existing approaches, ranging from rigid template matching to fully supervised learning, often fall short in real-world scenarios marked by protein heterogeneity, sparse annotations, and variable imaging conditions. Template-based methods tend to over-pick by misclassifying noise as particles, while deep learning models require extensive labeled datasets and struggle to generalize in low-data settings. These constraints have limited access to automation, particularly for novel proteins or resource-constrained labs. To address these challenges, we introduced CryoFSL, a few-shot learning framework combining SAM2 with lightweight adapter modules for efficient, robust particle picking with minimal supervision. CryoFSL outperformed traditional and deep learning models, not just in average metrics, but in performance stability across diverse proteins and imaging conditions. Remarkably, it performed well with as few as five annotated micrographs, maintaining high recall and F1 score regardless of annotation density. This level of resilience underscores the strength of its few-shot learning design and its ability to modulate features effectively for novel proteins.

The robustness of CryoFSL stems from its architectural design, which enables hierarchical feature modulation across multiple stages, adjusting to both local texture patterns (e.g. differentiating particles from ice crystals) and global context (e.g. resolving ambiguities), rather than relying on fixed correlation thresholds used by traditional methods. In contrast to the high variance seen in traditional methods, evidenced by wide interquartile ranges and outliers, CryoFSL achieves tight metric distributions, with a recall success rate of 96.6% at a threshold of 0.6 and 69.1% at 0.8, directly correlating with improved 3D reconstructions.

Notably, CryoFSL maintains near-optimal resolution even with only 10% labeled particles, outperforming baselines that used significantly more data. Visual comparisons confirm that CryoFSL avoids CrYOLO’s under-picking, Topaz’s redundancy, and template methods’ over-picking, achieving balanced and consistent outputs. CryoFSL’s intelligent selectivity enables superior 3D reconstruction resolution (average 5.33 Å) with significantly fewer particles than traditional methods, which select two to three times more particles. Though factors such as particle orientation and conformational heterogeneity may also contribute to observed resolution differences, precision in particle selection is often more critical than quantity, particularly when excess particles represent noise rather than structural diversity. This is supported by EMPIAR-10345, where CryoFSL’s 21 008 particles yielded a 3.84 Å resolution, outperforming baselines by ˃1.44 Å. Progressive reconstruction analysis additionally shows that even 25% of CryoFSL’s picks yield reconstructions comparable to complete sets of baseline outputs, underscoring the impact of high particle quality over quantity.

The strength of CryoFSL lies in resolving long-standing trade-offs in particle picking. Template-based methods achieve high recall via over-picking but suffer poor precision due to FPs, while deep learning models like Topaz and CrYOLO show unstable behavior under sparse annotations. In contrast, CryoFSL delivers consistently high performance across metrics by combining few-shot learning with efficient model adaptation. Its ability to achieve optimal results with just five annotated micrographs addresses the annotation bottleneck, and its lightweight adapter design ensures computational efficiency. Most importantly, CryoFSL generalizes reliably from minimal data, bridging the gap between automation and accuracy that has constrained traditional approaches.

Despite these advances, CryoFSL has limitations that merit consideration. In extremely low-data settings, such as 1-shot learning on complex proteins, the adapter may overfit to micrograph-specific noise patterns, limiting generalizability. This is seen in EMPIAR-11056, where the 1-shot configuration yielded F1 scores of 57.96%, with precision dropping as low as 49.97%, reflecting the difficulty under high heterogeneity. Representative examples illustrating the differences between 1-shot and 5-shot configurations are shown in Supplementary Figs S11 and S12. Similarly, very low contrast or degraded SNR can render the particle signal indistinguishable from the background, as observed in EMPIAR-10345, where even the 5-shot model achieved a precision of only 38.57%. Postprocessing assumptions (e.g. circularity, area) may fail under heavy aggregation or irregular shapes, leading to merged or missed picks; however, these are limitations of the postprocessing itself, not the adapter itself. Large domain shifts (e.g. uncommon contaminants or imaging conditions absent in training sets) can also misalign the frozen backbone’s priors, requiring a few additional labeled samples for correction. Future improvements could include domain-specific pretraining, active learning to select optimal training samples, and more adaptive postprocessing to better handle overlaps. Adopting efficient feature preservation strategies from a recent segmentation network could further enhance performance in a low-contrast scenario [55]. Extending CryoFSL to downstream tasks like particle classification or heterogeneity analysis could further enhance its impact within the cryo-EM pipeline. Moreover, the high-resolution structures enabled by accurate picking contribute to broader precision medicine efforts, complementing genomic-level machine learning applications for understanding disease mechanisms [56].

In conclusion, CryoFSL redefines data efficiency and generalizability in cryo-EM particle picking when few annotated micrographs are available. By combining the strengths of foundational vision models with the flexibility of few-shot adaptation, it bridges the gap between precision and practicality—enabling robust particle identification with minimal annotations, strong generalization across proteins, and higher downstream reconstruction quality. This represents a pivotal step toward scalable and reliable cryo-EM analysis in real-world research environments.

**Key Points**

- Cryo-EM Few Shot-Learning (CryoFSL) is a Segment Anything Model 2-based framework that achieves superior particle picking using only five annotated micrographs, reducing annotation burden while maintaining state-of-the-art accuracy, even for novel protein targets.
- It achieves robust performance with high precision and recall, excelling on noisy, diverse datasets and ensuring high-quality particle selection in computationally constrained environments.
- CryoFSL produces high-resolution 3D density maps using fewer particles, surpassing traditional and existing deep learning-based models.

## Supplementary Material

CryoFSL_Supplementary_materials_bbag285

## Data Availability

The dataset for this study is available on https://github.com/BioinfoMachineLearning/cryoppp. The source code is available at https://github.com/biplabpoudel25/CryoFSL.
